# Evaluation of DBS computational modeling methodologies using in-vivo electrophysiology in Parkinson’s disease^☆^

**DOI:** 10.1016/j.brs.2025.10.022

**Published:** 2025-10-27

**Authors:** Seyyed Bahram Borgheai, Bryan Howell, Faical Isbaine, Angela M. Noecker, Enrico Opri, Benjamin B. Risk, Cameron C. McIntyre, Svjetlana Miocinovic

**Affiliations:** a Department of Neurology, Emory University, Atlanta, GA, USA; b Department of Biomedical Engineering, Duke University, Durham, NC, USA; c Department of Neurosurgery, Emory University, Atlanta, GA, USA; d Department of Biomedical Engineering, University of Michigan, Ann Arbor, MI, USA; e Department of Biostatistics and Bioinformatics, Emory University, Atlanta, GA, USA; f Department of Neurosurgery, Duke University, Durham, NC, USA; g Department of Biomedical Engineering, Emory University and Georgia Institute of Technology, Atlanta, GA, USA

**Keywords:** Hyperdirect pathway, Corticospinal tract, Cortical evoked potential, Driving force, Volume of tissue activated, Normative space

## Abstract

**Background:**

Optimizing deep brain stimulation (DBS) parameter settings requires postoperative adjustments through a time-consuming trial-and-error process. As such, researchers have been developing computational models to guide DBS programming. Despite growing interest in image-guided DBS technology, and recent adoption into clinical practice, the direct validation of the prediction accuracy remains limited.

**Objective:**

The objective of this study was to establish a comparative framework for validating the accuracy of various DBS computational modeling methodologies in predicting the activation of clinically relevant pathways using in vivo measurements from PD patients undergoing subthalamic (STN) DBS surgery.

**Methods:**

In this study, we compared the accuracy of six computational modeling variations for predicting the activation of the corticospinal/bulbar tract (CSBT) and cortico-subthalamic hyperdirect pathway (HDP) using very short- (<2 ms) and short-latency (2–4 ms) cortical evoked potentials (cEPs). We constructed the variations using three key factors: modeling method (Driving Force [DF] vs. Volume of Tissue Activated [VTA]), imaging space (native vs. normative), and anatomical representation (pathway vs. structure). The model performances were quantified using the coefficient of determination (R^2^) between the cEP amplitudes and percent pathway or structure activation.

**Results:**

We compared model accuracy for 11 PD patients. The DF-Native-Pathway model was the most accurate method for quantitatively predicting experimental subcortical pathway activations. Additionally, our analysis showed that using normative brain space significantly diminished the accuracy of model predictions.

**Conclusion:**

The choice of methodology should depend on the specific application and the required level of precision for the intended analysis. However, model parameters should be optimized to accurately predict known experimental activation measures.

## 1. Introduction

Deep brain stimulation (DBS) is an effective therapy for Parkinson’s disease (PD) and is being explored for various neuropsychiatric disorders. However, DBS efficacy depends on complex and time-consuming trial-and-error postoperative programming, further complicated by technological advances like segmented leads that expand the parameter space [1,2]. Computational DBS models are of increased interest to address these limitations through in-silico approaches, reducing the need for continuous patient interaction. These 3D, image-based, biophysical models visualize neuronal activation patterns around electrodes and connected regions [3]. Pilot studies suggest they can reduce programming time [4,5] and improve outcomes [6]. Modeling DBS effects in 3D is also valuable for understanding neural pathways and mechanisms involved in therapeutic effects and side effects [7–11].

Despite growing interest in image-guided DBS programming and recent adoption into clinical practice, the direct validation of model prediction accuracy remains limited. For instance, studies correlating model-defined ‘sweet spots’ with clinical outcomes have shown statistical significance but low correlation [12–15] and the models are generally poor at predicting stimulation-induced side effects [16]. In addition, wide-ranging variability in the technical modeling methodologies translates into a lack of standardization that has hindered validation [17,18]. To make the use of DBS models justifiable in clinical research, and successful in clinical practice, a rigorous and systematic optimization and validation is necessary to demonstrate that DBS models accurately estimate activation of related structures and pathways [19–21].

In this study, we focus on comparing three key sources of variability in DBS modeling: imaging space, biophysical modeling method, and anatomical representation. Patient-specific pipelines typically utilize native imaging space to define individual anatomy, electrode placement, and pathway trajectories [19,20]. Alternatively, co-registering patient imaging into a normative space enables the use of standardized atlases, facilitating group-level analyses like probabilistic “sweet spot” mapping for therapeutic efficacy [12,13,21,22]. Biophysical modeling of neuronal activation around the stimulating contact also differs in complexity. Simplified volume of tissue activation (VTA) models estimate activation using uniformly distributed axons around the electrode [23] and fixed electric field thresholds as proxies for axonal activation in symmetric/isotropic fields [24–26]. Driving force (DF) models, in contrast, leverage electric field gradients and more realistic axonal trajectories derived from tractography or pathway atlases [19,27]. Anatomical representation is another factor distinguishing DBS modeling. VTA models traditionally use structure-based atlases, assuming activation overlaps with target regions, while DF models and more recent probabilistic VTA approaches (coupled with fiber filtering) incorporate pathway (connectomic) atlases to account for individual axons passing near the electrode [28–31]. Despite these advancements, no comparative studies have definitively determined which imaging space, modeling method, or anatomical representation provides superior accuracy or clinical utility. Moreover, since many studies [32–36] continue to rely on simpler VTA models with default settings in commonly available tools, and apply them in normative space, it is important to experimentally evaluate the accuracy of such approaches. In addition, there is a need to investigate whether more complex models with optimized parameters improve predictive power [29]. Specifically, our goal was to determine whether transitioning from normative to patient-specific space, from default VTA-based models to optimized DF-based models, or from structure-based anatomy to pathway-based anatomy, would result in higher predictive accuracy.

To address these questions, we established a comparative framework for assessing the accuracy of various DBS computational modeling methodologies in predicting the activation of clinically relevant pathways using in vivo measurements from PD patients undergoing subthalamic (STN) DBS surgery. We focused on the hyperdirect pathway (HDP) — a direct projection from the cortex to the subthalamic nucleus (STN) formed primarily by thin collaterals of corticofugal axons passing through the internal capsule. This pathway is hypothesized to be involved in DBS therapeutic benefit [37–40]. Additionally, we examined the corticospinal/bulbar tract (CSBT), whose activation results in unwanted muscle contractions which is one type of DBS side effects [41].

**Fig. 1.**
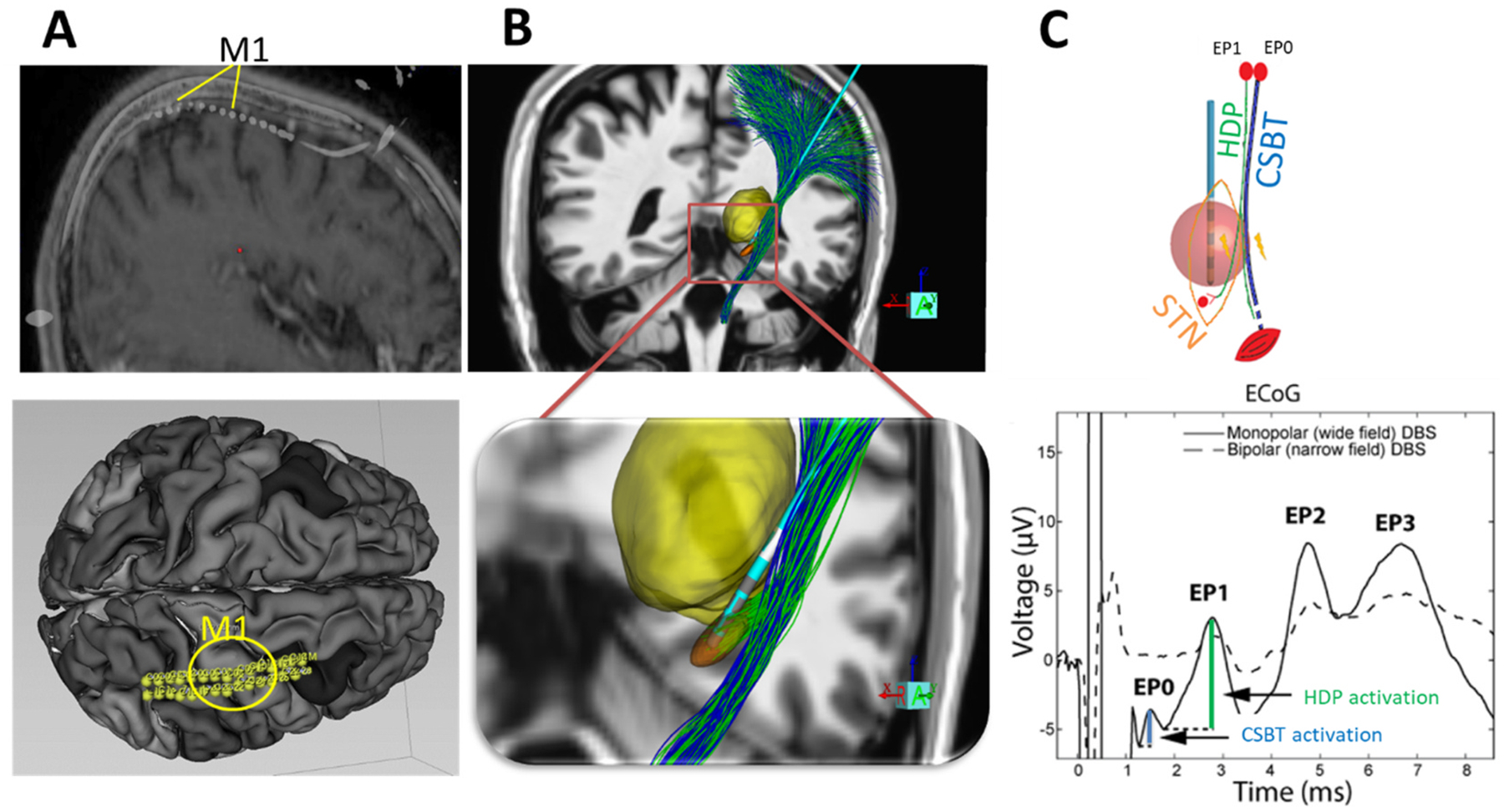
Experimental Setup and Target Pathways. A) Top: Sagittal view of a temporary 28-contact subdural electrocorticography (ECoG) strip placed over the M1 region. Bottom: Top-down view of the ECoG placement visualized by Slicer 5.2. B) Modeling of the DBS electrode implanted in the subthalamic nucleus (STN) (orange), shown in relation to the thalamus (yellow), the hyperdirect pathway (HDP, green), and the cortico-spinal bulbar tract (CSBT, blue), visualized by StimVision. C) Top: Schematic representation of HDP and CSBT antidromic activation in response to STN stimulation. Bottom: Example of a cortical evoked potential (cEP) recorded from one of the ECoG strip contacts in response to STN stimulation. The first and second peak amplitudes, referred to as very short-latency evoked potential (EP0) and short-latency evoked potential (EP1) [42], are used here as measures of HDP and CSBT activation. STN = subthalamic nucleus; HDP = hyperdirect pathway; and CSBT = corticospinal/bulbar tract.

We employed cortical evoked potentials (cEPs) as an objective gold standard of pathway activation, as previous studies have shown that the latencies and amplitudes of cEPs evoked by DBS can reveal which pathways near the STN are activated [40,42,43]. Specifically, EP0 is the earliest (<2 ms), it occurs primarily with high intensity stimulation and is accompanied by concurrent activation of peripheral muscles therefore consistent with activation of the fast-conducting CSBT [42]. The latency of EP1 is longer at 2–4 ms, it is easily elicited with DBS contacts in the STN and therefore consistent with activation of thin HDP collaterals [42,44]. We used these experimental measures to assess the accuracy of different DBS modeling methodologies in predicting activation of CSBT and HDP (Fig. 1C). Our findings aim to guide the development of more accurate models in both DBS clinical practice and research.

## 2. Materials and methods

### 2.1. Patient selection

Patients with idiopathic PD scheduled for awake STN DBS surgery at two major academic centers participated in the study (Table 1). Written informed consent was obtained preoperatively under the Institutional Review Boards approved protocols. Patients were informed that a temporary subdural electrocorticography (ECoG) recording electrode would be inserted exclusively for research purposes during the procedure.

### 2.2. Experimental setup and signal acquisition

In this study, we used the same experimental dataset reported in our previous work [29]. Briefly, cortical evoked potentials (cEP) were recorded with a subdural ECoG strip (28 or 6 contacts; Ad-Tech) placed on the surface of the brain via the same burr hole used for DBS implantation [42,45] (Fig. 1-A). Recordings were performed at least 12 h after stopping all anti-parkinsonian medications and at least 30 min after stopping propofol sedation. ECoG electrode location was determined using intraoperative CT registered to the preoperative MRI in surgical planning software (Framelink 5.1, Medtronic). Contact pairs overlying the precentral gyrus (M1) were used for further analysis (Fig. 1-A). ECoG potentials were recorded using either the Neuro Omega (Alpha Omega Engineering) at a sampling rate of 22 kHz or TDT PZ5 (Tucker Davis Technologies; P09 and P11) at a sampling rate of 24,414 Hz. Ear electrode (P01-P06) or an ipsilateral scalp needle (P07-P11) was used as the reference with the corresponding contralateral electrode as the ground.

### 2.3. Experimental stimulation and recordings

We experimentally measured antidromic activation of the hyperdirect pathway (HDP, green) and the corticospinal-bulbar tract (CSBT, blue) generated by STN DBS (Fig. 1B and C, top). The number of stimulation settings tested varied across patients (range 15–49) due to logistical constraints and available intraoperative time (Table 1). We used the same datasets and stimulation settings as in our previous study [29]. All stimulation waveforms (except for three settings) were asymmetric biphasic, with a 70 μs inter-phase interval (Fig. 1C, top), and were applied for 12 s at 10 Hz resulting in ~120 pulses. The stimulation settings (360 total) were monopolar (cathodic) or bipolar, with amplitudes ranging from 1 to 5 mA, a pulse width of 60 μs (except for 12 settings at 30 or 120 μs), and a frequency of 10 Hz (except for 8 settings at 130 Hz). Detailed neuroimaging and lead localization methods are described in the supplementary material.

### 2.4. Computational modeling variations

**Fig. 2.**
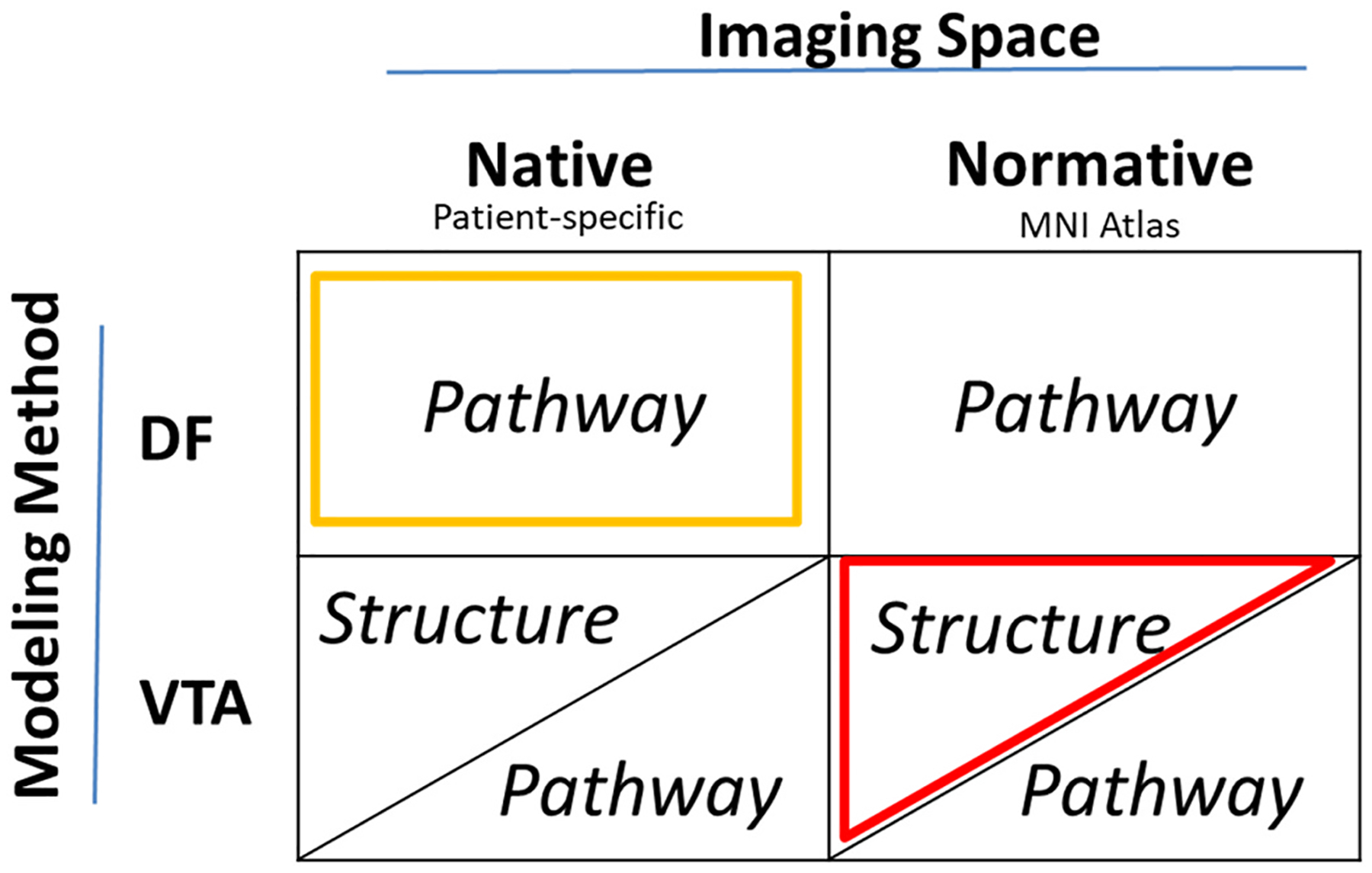
Computational model variations based on three factors: Modeling methods (VTA or DF), Imaging space (Native or Normative), and Anatomical representation (Structure or Pathway). DF by definition only models pathway activations, so there is no DF with Structure-based atlases. The highlighted models (in yellow and red) are the most commonly used in published modeling studies.

We implemented six model variations based on combination of three key factors: imaging space, modeling method, and anatomical representation (Fig. 2). The modeling method was either DF or VTA. The imaging space was either native (patient-specific) or normative (atlas-based). Neural activations in VTA-based models were calculated using either volumetric structures (overlap of anatomical structure with the VTA) or pathway streamlines (number of neural streamlines that pass through the VTA). For DF-models, pathways were the only viable anatomical representation due to their inherent focus on axonal activation predictions. We therefore created 6 model variations: 1) DF-Native-Pathway, 2) DF-Normative-Pathway, 3) VTA-Native-Structure, 4) VTA-Normative-Structure, 5) VTA-Normative-Pathway, and 6) VTA-Native-Pathway.

### 2.5. Modeling methods

#### 2.5.1. Volume of Tissue Activation (VTA)

**Fig. 3.**
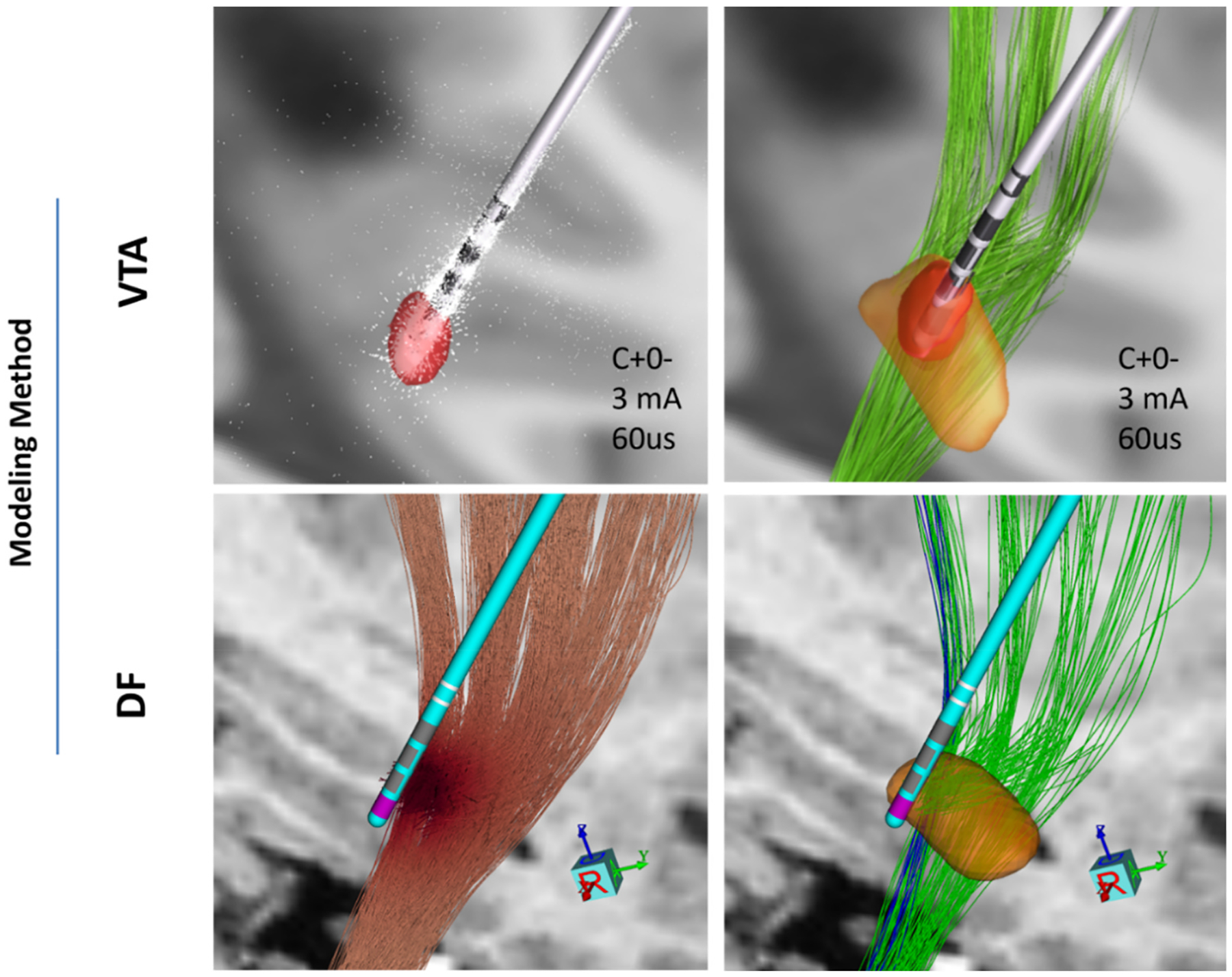
Example of different modeling methods for one patient (P06): Top images are generated in Lead DBS V3.1 and bottom ones in StimVision. Top-Left: Volume of Tissue Activated (VTA) estimate in red in response to monopolar stimulation at ventral-most contact (gray). White arrows represent electric field lines; Top-Right: VTA overlap with STN structure (orange) and HDP (green); Bottom-Left: Driving Force (DF) estimate of voltage distribution gradients along the axonal streamlines; Bottom-Right: Estimate of activated pathway streamlines for HDP (green) and CSBT (blue) in response to the same stimulation setting as above.

The VTA is a binary volume surrounding the stimulating contact, predicted to activate axons and elicit action potentials within it [46]. We used Lead DBS v3.1 to construct VTAs around the stimulating leads using their default parameters including conductivity of white and gray matter as 0.14 S/m and 0.33 S/m, respectively, with ‘electrode removed’ option checked. Lead DBS uses the atlas (in our case Distal atlas) to differentiate white and gray matter. That software employs the SimBio/FieldTrip pipeline [47] with the finite element method (FEM) to solve the Laplace equation in discretized native patient space (Fig. 3, Top-Left). The default threshold of 0.2 V/mm was applied to the E-Field magnitude to create the VTA [24,26,48] which was adopted by Lead DBS based on Astrom et al. work (2015) [24] suggesting this threshold with the pulse width as 60 us and the fiber diameter as 3.5 μm. Since the software, in its default setting, does not account for stimulation frequency or pulse width differences, changes in these parameters produced the same VTA. We used the standard GUI in Lead DBS to input stimulation settings which let the user input amperage, monopolar and bipolar contact settings with the default biphasic waveform assuming 60 us pulse width. The VTAs were then used to calculate the percentage overlap with atlas structure volume or to calculate the number of pathway streamlines passing through the VTA as a measure of neural activation (Fig. 3, Top-Right).

#### 2.5.2. Driving force (DF)

DF predictors were constructed as described in our prior study [29]. Electric field models were built in COMSOL (v5.1). The volume conductors for DBS leads were modeled with Dirichlet and mixed boundary conditions for active and inactive contacts, respectively. Tissue models for standard and steerable leads were discretized in 2D and 3D, respectively, for efficiency. 2D axisymmetric models were used to model DBS with standard leads due to their axial symmetry. 3D models were used for directional leads. Electric potentials were calculated by solving Laplace’s equation using the Finite Element Method (FEM). Temporal variations were approximated with a waveform derived from an equivalent circuit model [29,49] in which the DF model is linear, so the spatiotemporal potential field can be approximated as the potential field at t = 0 multiplied by the voltage drop across a 1D equivalent circuit of the volume conductor model. We used a Floating Potential boundary condition for passive electrodes and enforced two conditions on the boundaries: 1) all potentials are equal, and 2) the net current flow is zero. We validated the use of this method in a prior work [50]. The 0.5 mm-thick scar surrounding the model lead had a conductivity (S_scar_) of 0.07 S/m. The bulk tissue medium was homogeneous and isotropic with a conductivity (S_tissue_) of 0.2 S/m. Axonal stimulation thresholds were estimated using a predictive algorithm based on the driving force at the nodes of Ranvier within the CSBT and HDP [27].

### 2.6. Imaging space

**Fig. 4.**
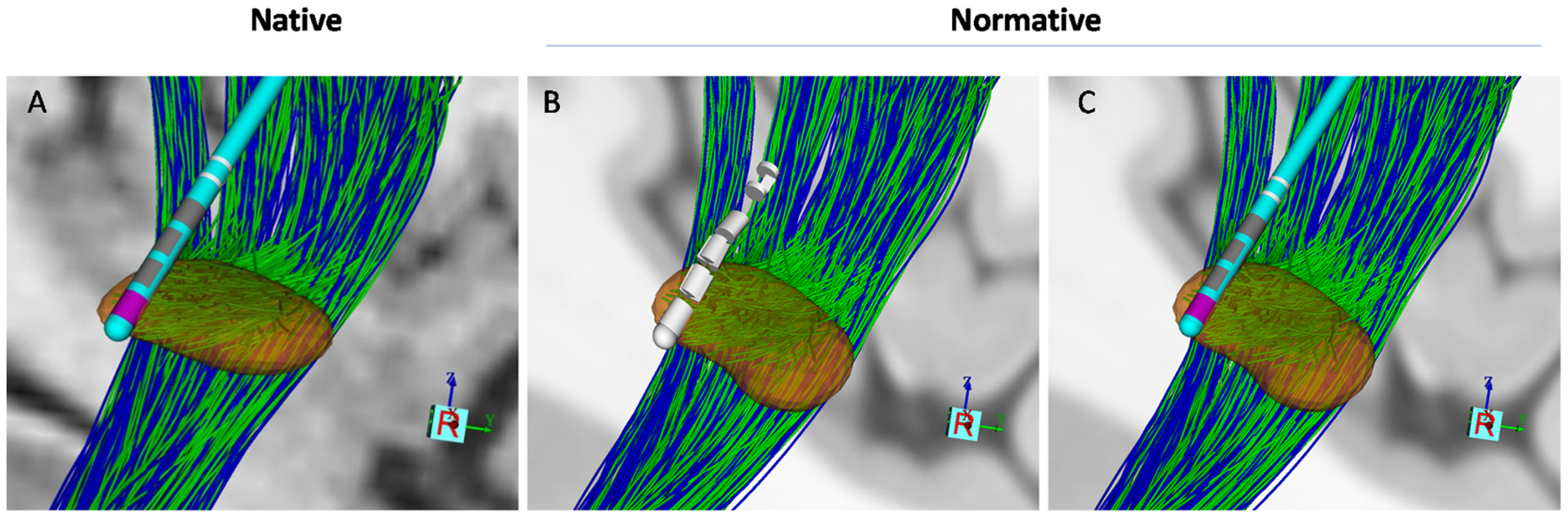
Example of modeling in different imaging spaces for one patient (P06). The normative space used here is MNI2009b. All images are generated in StimVision. The lead type is ABT6172, and STN, HDP, and CSBT are shown in orange, green, and blue, respectively. Transformation into normative space introduces visually subtle warping of the anatomical structures and the lead shaft. A) Illustration of the STN, lead, and pathways in native space, B) Warped image in normative space before fitting a lead shaft to a straight line between the contacts and tip. C) Warped image in normative space after fitting a lead shaft to a straight line between the contacts and tip.

Each modeling method was implemented in either native (patient-specific) or normative (atlas-based) space (Fig. 4).

#### 2.6.1. Native

For DF native models, the standard CIT168 [51] brain atlas was registered to the patient’s preoperative T1-weighted space using FSL’s nonlinear registration tool, FNIRT [52], with all registrations visually confirmed for quality. Additionally, CSBT and HDP were modeled using an anatomical pathway atlas of the subthalamic region (Fig. 3) defined in the CIT168 space [53] and individualized by nonlinearly warping the atlas data from CIT168 space to the patient’s preoperative T1w (native) space.

For VTA native models, the VTAs were first calculated in native space using the SimBio/FieldTrip pipeline [47] in Lead DBS v3.1 which is the static formulation of the Laplace equation solved in native patient space.

#### 2.6.2. Normative

For normative space, to provide a common ground between DF and VTA methods, all the imaging and atlases were warped into ICBM 2009b Nonlinear Asymmetric space known as Montreal Neurological Institute (MNI) space [54]. This version of normative space was the updated version of MNI space in 0.5 × 0.5 × 0.5 mm^3^ resolution constructed through nonlinear co-registration of 152 acquisitions [26,54].

### 2.7. Anatomical representation and activation measures

Two anatomical representations were used when estimating the effects of DBS: Pathway or Structure.

#### 2.7.1. Pathway

**Fig. 5.**
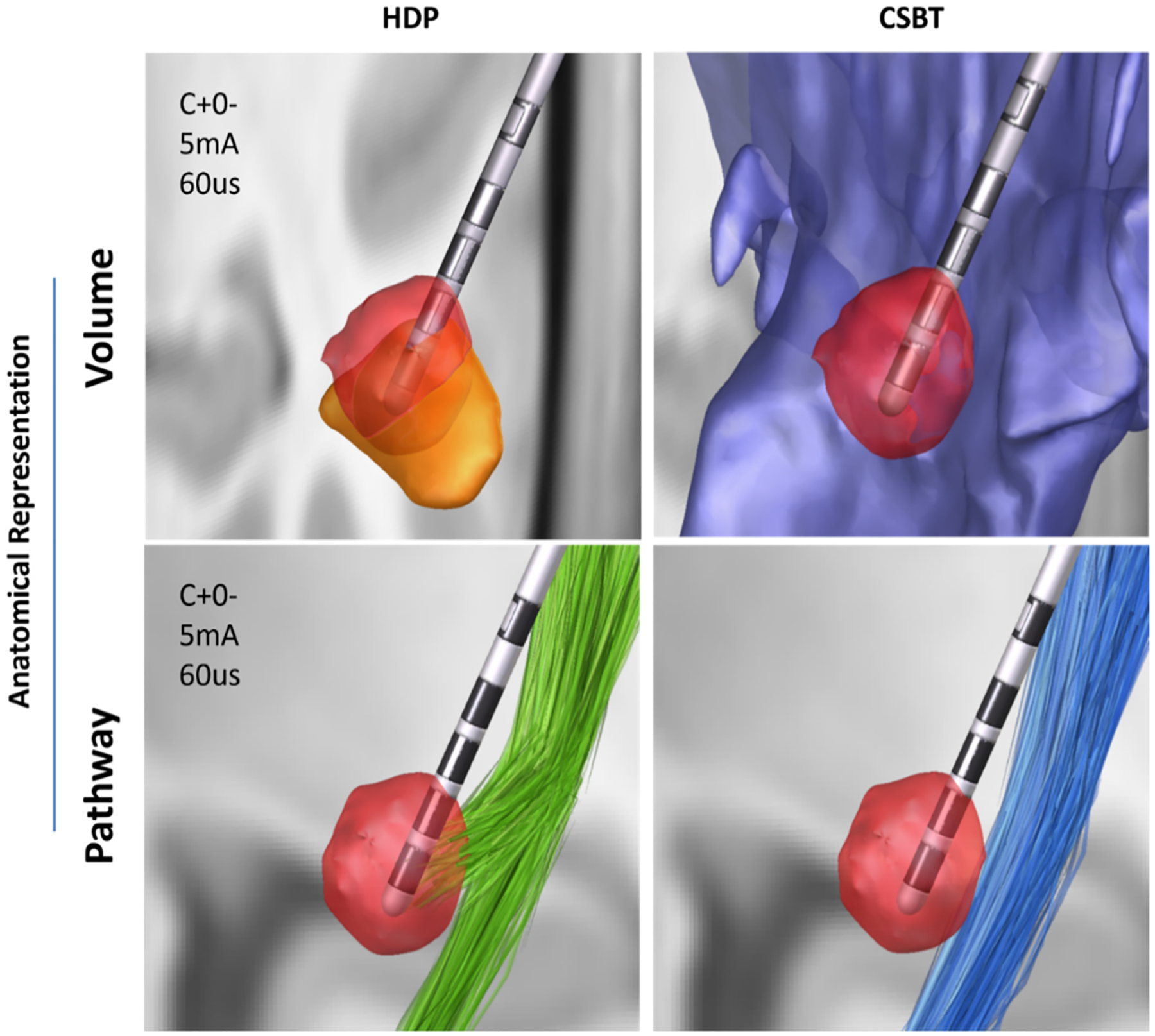
Example of different anatomical representations for one patient (P06). Top row illustrates the structure representation using Distal atlas showing overlaps of a VTA with STN (orange-Left) and IC (purple-Right). Bottom row illustrates the intersection of a VTA in response to the same stimulation with HDP (in green-Left) and CSBT (in blue-Right).

The same pathway atlas was used for VTA and DF models previously described by Petersen et al. [53]. For DF models, each pathway fiber whose activation threshold was below the stimulation amplitude was counted as activated by that stimulation setting. The percentage of fibers activated for HDP and CSBT was used as a metric of activation for each pathway (Fig. 3- Bottom-Right). We set fiber diameters as 12 μm for CSBT and 4 μm for HDP based on our prior work which showed this was the optimal diameter to accurately model experimental activations [29]. The reason for 12 μm fiber diameters to have the optimal accuracy could be that a small minority of large fibers [55] is largely mediating the EPs, so percent activation reflects activation of this subset of fibers rather than the whole pathway, because most brain fiber bundles are comprised of small (<4um) fibers [56]. Moreover, DF predictor, though it includes more realistic biophysical details, still uses a simplified volume conductor with isotropic tissue conductivity which leads to over-estimated axonal activation thresholds compared to models with anisotropy and heterogeneity [50]. This latent hypoexcitability can be correted by modeling larger fibers, but the reality may be that relatively smaller fiber diameters (e.g., 6–9 μm) are actually mediating the EPs. For VTA models, we calculated the percentage of fibers passing through VTAs using custom MATLAB code described in the supplementary material (Fig. 5- Bottom). To generate VTA-Native-Pathway model, we warped the Peterson atlas into patient specific (native) space for each subject. Because the native space in Lead-DBS is reoriented relative to the original preoperative T1 image, we first co-registered the reoriented preoperative T1 in Lead-DBS to the original preoperative T1 and then applied the resulting transformation to all VTAs constructed in Lead-DBS native space.

#### 2.7.2. Structure

The overlap of VTAs with internal capsule (IC) and STN structures in the DISTAL atlas [57] were calculated as activation metrics for CSBT and HDP, respectively, in both normative (MNI) and native spaces (Fig. 5-Top). In the normative space, we calculated the percentage of overlap volume with respect to the desired structure volume (i.e. IC and STN), using the voxel-based values reported in Lead-DBS [26,48]. For the overlaps in the native space, we developed a custom MATLAB code where we calculated the percentages of the structure (IC or STN) vertices located inside VTA objects in response to each DBS setting (as detailed in the supplementary material).

### 2.8. Statistical analysis

The model performance was quantified using the coefficient of determination (R^2^), i.e., the square of Pearson correlation coefficient (R), between the experimental measures of HDP and CSBT pathway activation (i.e., cEP amplitudes) and the corresponding estimates of pathway activation produced by each computational model. The predictive accuracy of all modeling variations was assessed relative to the DF-Native-Pathway described in our previous work [29] using Wilcoxon signed-rank test to compare the corresponding R^2^ values across all patients for the HDP and CSBT pathways, with a significance level of α = 0.05. We first compared the two most common modeling strategies in the DBS literature: DF-Native-Pathway and VTA-Normative-Structure. Next, to investigate the influence of different modeling factors (imaging space, modeling method, and anatomical representation), we performed comparisons using the Wilcoxon signed rank on R^2^ values of two models that were identical in all aspects except for the factor under investigation.

For the CSBT pathway, in addition to R^2^, we compared different models using F-score values, based on presence or absence of activation, to account for the imbalance in the number of true negatives in our data. This imbalance resulted from the presence of numerous zero values in either the model’s estimated values or the EP0 amplitudes —on average, 76.8 % of EP0 amplitudes were zero per patient (range: 30–100 %). In F-score calculation, we excluded three patients whose EP0 was equal to zero for all stimulation settings.

## 3. Results

We compared the performance of different DBS computational models in predicting HDP and CSBT activations using cortical evoked potentials from 11 PD patients with STN-DBS as the experimental activation metric (the group visualization of lead reconstructions is illustrated in Fig. S1). In each patient, for each stimulation setting (360 total-Table 1), we estimated the activation of HDP and CSBT pathways using six model variations: DF-Native-Pathway, DF-Normative-Pathway, VTA-Normative-Pathway, VTA-Normative-Structure, and VTA-Native-Structure. The average number of stimulation settings tested and modeled per patient was 32 (range: 15–49) (Table 1). The model performance was evaluated using the coefficient of determination (R^2^) to quantify the relationship between measured cEP amplitudes and model predictions (either percent pathway activation or activation volume overlap), for two pathways of interest (HDP and CSBT).

**Fig. 6.**
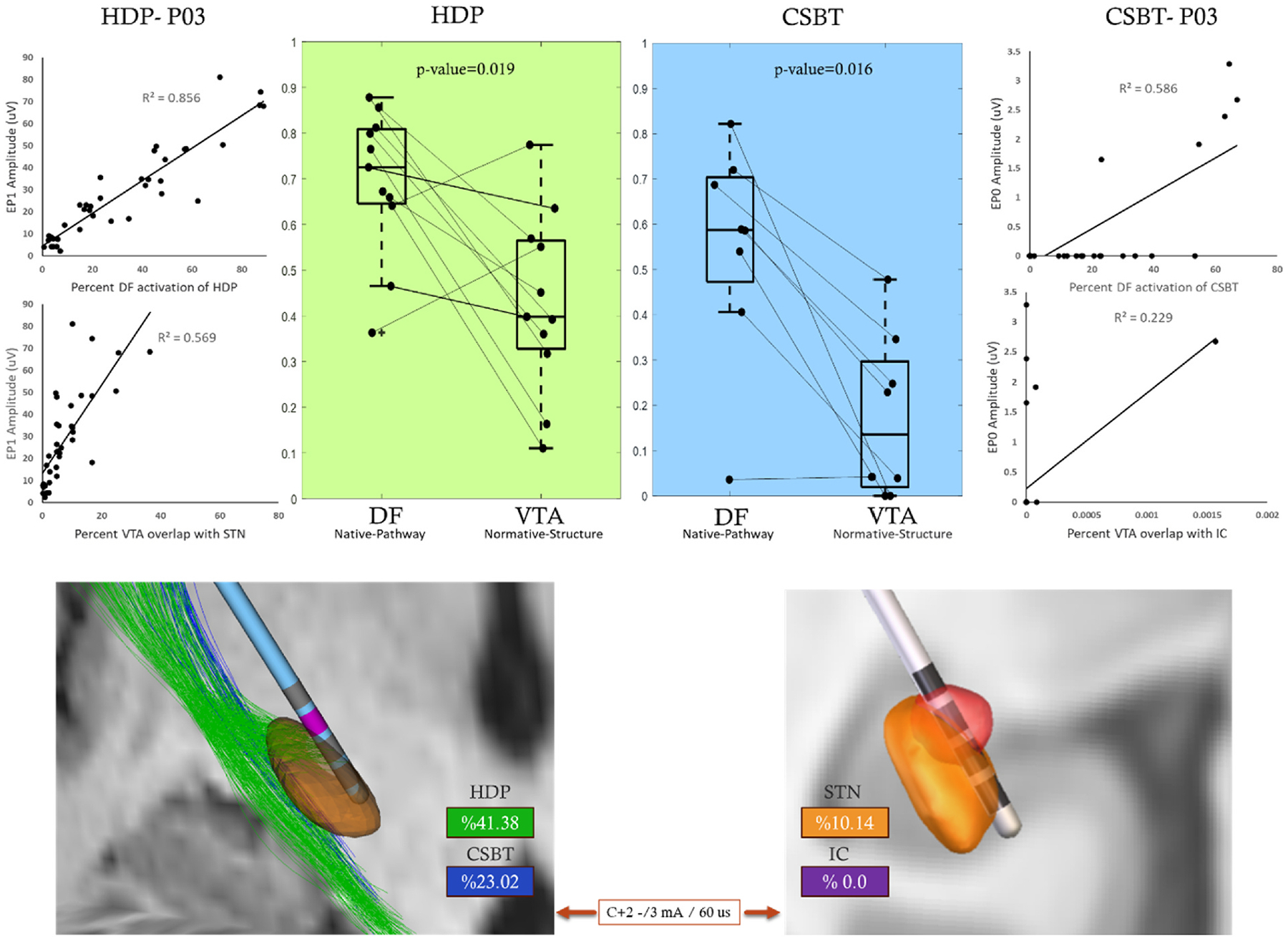
Comparison between the predictive performance of two common model types: DF-Native-Pathway and VTA-Normative- Structure for two pathways of interest: HDP (green-left) and CSBT (blue-right). Top: Each data point in a box plot is the R2 value for one patient, i.e. the square of the correlation coefficient between the modeling activation predictions and experimental cEP amplitudes. Example correlation plots are shown for one patient (P03) comparing model predictions and cEP amplitudes for all stimulation settings. Bottom: An example of DF-based prediction of pathway activations (Left) versus VTA-based prediction of overlap with STN structure (Right) in the same patient (P03). Internal capsule structure is not shown on the right for clarity.

First, we compared the two most commonly used model variations, DF-Native-Pathway and VTA-Normative-Structure (Fig. 6). The median model performance was higher using the DF method, for both pathways. For HDP, the *R*^2^ was 0.73 (IQR: 0.65–0.81) for DF and 0.40 (IQR: 0.33–0.57) for VTA. This difference was statistically significant (p-value = 0.019). For CSBT, the *R*^2^ median was 0.59 (IQR: 0.47–0.70) for DF and 0.14 (IQR: 0.02–0.30) for VTA. This difference was statistically significant (p-value = 0.016). The VTA model performance was particularly poor for estimating the activation of CSBT pathway (modeled as overlap between the VTA and internal capsule structure). For CSBT, F-score median was 0.77 (IQR: 0.35–0.86) for DF and statistically comparable at 0.55 (IQR: 0.42–0.80) for VTA (p-value = 0.945) (Fig. S2). Overall, DF-Native-Pathway model performed better than all other models across all patients (Tables S1 and S2). In three patients with directional leads (P04, P05, and P06), to see the impact of directional stimulation on model performance, we compared the performance of two conventional models, separately for directional (segmented contact) and non-directional (ring or pseudo ring) settings (the formal statistical comparison was not performed given only 3 subjects). In DF conventional method (DF-Native-Pathway), in both HDP and CSBT, the model performance worsened when only directional settings were considered. In VTA conventional method (VTA-Normative-Structure), HDP predictions worsened for directional settings, but it improved in CSBT. However, in both pathways, the DF conventional method performance medians remained higher compared to VTA conventional method for both directional and non-directional settings (Fig. S3).

**Fig. 7.**
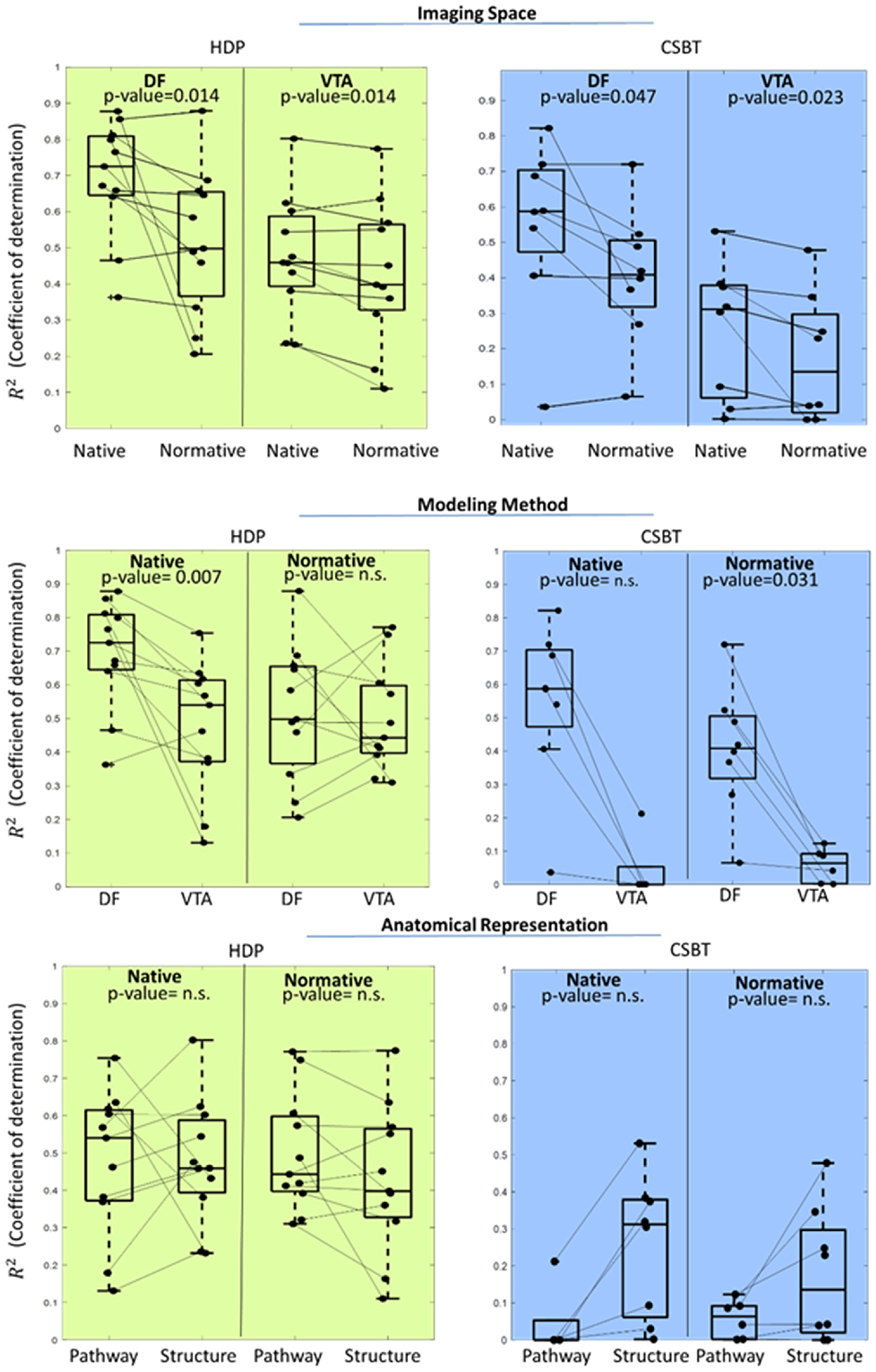
The effect of three key factors on model performance when predicting activation of HDP (green-left) and CSBT (blue-right) pathways. Nonsignificant differences are shown by n.s. Top: Two model types – DF-Pathway and VTA-Structure were generated in native and normative space and in all cases the native model outperformed its normative counterpart. Middle: DF and VTA modeling methods were directly compared in both spaces. DF outperformed VTA in predicting CSBT activation. Bottom: Pathway and Structure anatomical representation were compared in VTA models with no significant differences.

We compared the models based on three factors to determine how each contributed to the observed performance differences (Fig. 7). To assess the importance of imaging space, we compared methodologies which were built in both native and normative spaces which resulted in two paired comparisons in each pathway: 1) DF-*Native*-Pathway versus DF-*Normative*-Pathway; 2) VTA-*Native*-Structure versus VTA-*Normative*- Structure. For HDP, the median *R*^2^ was 0.73 (IQR: 0.65–0.81) and 0.46 (IQR: 0.39–0.59) for DF-Pathway and VTA-Structure in native space, respectively, and both were significantly higher compared to 0.50 (IQR: 0.37–0.66) (p-value = 0.014) and 0.40 (IQR: 0.33–0.57) (p-value = 0.014) for their respective models in normative space. For CSBT, the *R*^2^ median was 0.59 (IQR: 0.47–0.70) and 0.31 (IQR: 0.06–0.38) for DF-Pathway and VTA- Structure in native space, respectively and both significantly higher compared to 0.41 (IQR: 0.32–0.51) (p-value = 0.047) and 0.14 (IQR: 0.02–0.30) (p-value = 0.023) for respective modeling methodology in normative space (Fig. 7-Top).

To directly compare the effect of modeling method (DF versus VTA), we compared normative and native space with pathway representation. For HDP, in the normative space, the median *R*^2^ was 0.50 (IQR: 0.37–0.66) for DF and comparable to 0.44 (IQR: 0.40–0.60) for VTA model (Fig. 7-Middle/Left). In the native space, the median *R*^2^ was 0.73 (IQR: 0.65–0.81) for DF and comparable to 0.54 (IQR: 0.39–0.59) for VTA model (Fig. 7-Middle/Left) (p-value = 0.007). For CSBT, in the normative space. the *R*^2^ median was 0.41 (IQR: 0.32–0.51) for DF which was significantly higher compared to 0.06 (IQR: 0.00–0.09) (p-value = 0.031) for VTA model (Fig. 7-Middle/Right). In the native space, the median *R*^2^ was 0.59 (IQR: 0.47–0.70) for DF which was higher compared to 0.00 (IQR: 0.06–0.38) for VTA model (Fig. 7-Middle/Right). Also, the F-score metric showed significantly higher values for DF method with median of 0.52 (IQR: 0.34–0.72) compared to VTA at 0.10 (IQR: 0.00–0.28) (p-value = 0.008) (Fig. S2).

Finally, to evaluate the effect of anatomical representation (pathway versus structure) we used VTA models in normative and native space as the only possible pairs for comparison among the implemented variations. For HDP, the median *R*^2^ was 0.44 (IQR: 0.40–0.60) for Pathway model and comparable to 0.40 (IQR: 0.33–0.57) for Structure model (Fig. 7. Bottom/Right). For CSBT, the *R*^2^ median was 0.06 (IQR: 0.00–0.09) for pathway and comparable to 0.14 (IQR: 0.02–0.30) for Structure model (Fig. 7- Bottom/Left). These patterns were consistent in native space. The F-score metric for CSBT activation showed significantly higher values for the Structure model with median of 0.55 (IQR: 0.42–0.80) compared to Pathway model at 0.10 (IQR: 0.00–0.28) (p-value = 0.008) (Fig. S2).

## 4. Discussion

We compared activation predictions from six different computational DBS model variations -differing in biophysical modeling method (DF versus VTA), imaging space (native versus normative), and anatomical representation (Structure versus Pathway)- with experimental measures of HDP and CSBT pathway activations from PD patients undergoing STN DBS surgery. In general, DF models were more accurate than VTA models, although there were some cases such as Normative-Pathway methodology for HDP activation for which the performance of VTA and DF were comparable. Furthermore, predictions were more accurate in the native compared to normative space, independent of the other methodologies. Overall, the DF models implemented in native space with pathway representation most accurately predicted experimental activations compared to all other methodologies, including the conventional VTA models implemented in normative space with structure representation.

When we separated directional settings from non-directional ones in three patients with directional leads, we observed reduced performance for both DF (in both pathways) and VTA conventional models (in HDP). This reduction may result from the greater sensitivity of directional modeling to activation positioning, atlas accuracy, and tissue anisotropy and heterogeneity [29,58,59]. Surgical factors—such as the presence of air bubbles, cerebrospinal fluid loss, or changes in intracranial pressure due to pneumocephalus [60,61]—can further compromise imaging accuracy and, consequently, lead reconstruction. All these errors can be reflected more prominently in directional-setting modeling compared to full-ring modeling. Moreover, it is noteworthy that while the EPs were recorded at M1, we focused on the whole STN or all divisions of HDP or CSBT, rather than the motor STN (for Structure) or M1 divisions (for Pathway). One reason was that in our previous work [29], we achieved higher predictive performance when all divisions were included. This might be because the traditional tripartite view of the STN—dorsolateral motor, ventromedial associative, and medial limbic—likely over-simplifies the overlapping and heterogeneous topology of HDP and CSBT across patients. Additionally, the borders between different regions within STN are hypothetical and differ between atlases, and in this sense, it might be safer to consider the whole STN. We compared R^2^ for motor STN and whole STN, and the results were highly correlated (Fig. S4- Left). The absolute percent overlap of target structure and VTA differs between motor STN and whole STN, but they are proportional since motor STN is large and all leads are within it (Fig. S4- Middle) and therefore the correlation metric (R^2^) is almost unchanged. On the other hand, in the Peterson atlas, M1 HDP projects to a smaller (dorsal) region of the STN so model performance is more affected by choice of pathway division (Fig. S4- Right).

### 4.1. Modeling in native space improves overall predictive performance

Independent of the modeling method or anatomical representation used, our analysis demonstrated that implementing methodologies in patient-specific (native) space resulted in better performance in predicting the experimental measures of HDP and CSBT activation compared to normative space. The superior performance of DBS modeling in patient-specific space highlights the importance of constructing individualized models rather than relying on analyses performed within a generic normalized atlas space. Especially in VTA-based methods, the superiority of modeling in native space may be explained by the fact that while the non-linear transformation from patient space to normative space is applied to images and atlases (pathway or structure), the electric field is computed without incorporating this transformation. This observation may also explain why recent efforts to use modeling software to correlate model predictions in normative space with PD motor symptom improvement for individual patients have been largely unsuccessful [14], especially when employing modeling based on aggregate patient data [14,28,62]. A recent study also showed that incorporating native space in the methodological pipeline can improve correlations with clinical outcomes compared to normative space [63].

### 4.2. DF modeling has a performance advantage for CSBT activation predictions

The performance advantage of the DF-models compared to VTA-models was more pronounced when predicting CSBT(Δr~0.38) activation compared to HDP(Δr~0.21). Additionally, in normative space the difference between DF and VTA models was statistically significant only for CSBT(Δr~0.41). Several factors may have contributed to this result. First, VTA-based models can accurately estimate stimulation effects close to the electrode, but they underestimate activation of pathways or structures that are farther away [64]. This effect may be more pronounced by moving the analysis into the MNI normative space, which is an exceptionally large brain volume, resulting in CSBT streamlines residing even farther from the stimulating contact and resulting in fewer activated fibers. Using default parameters in Lead DBS (the electric field potential threshold as 0.2 V/mm assuming pulse width as 60 us and the fiber diameter as 3.5 μm), the corresponding VTA models underestimate the spread of stimulation effects whether using anatomical structure representation or a pathway atlas. This is likely because conceptually these models are similar-they calculate either how much structure or how many streamlines overlap with a fixed VTA.

VTA models may underestimate stimulation effects at larger distance from the electrode because either voltage field estimate falls off too quickly or the voltage field threshold used to generate VTA is suboptimal [64,65]. This means that it is necessary to optimize this threshold and related model parameters based on the application or pathway of interest. Specifically in the case of CSBT, a fundamental issue is that VTA algorithms are generic representations of stimulation spread, derived from a single fiber diameter in an isotropic brain medium. However, CSBT fibers are embedded in the highly anisotropic internal capsule, which alters the DBS voltage distribution [66] and substantially alters axonal response thresholds [50]. Alternatively, DF models account for the fact that voltage distribution ‘seen’ by the axons varies along their trajectory. These factors help explain why reports using patient-specific DBS electric field models, explicitly representing brain anisotropy [67,68] reported a stronger association between CSBT activation and clinical side effect thresholds compared to simpler models [16].

It should be noted that the VTA-based methods assessed in our work and many other DBS studies [32–36] were constructed using the default parameters in Lead-DBS—such as conductivity and fiber diameter—and in particular, it was not explicitly optimized to simulate the activation of internal capsule fibers. Some of these parameters differed from those used in the DF-based methods and so these discrepancies may have contributed to the observed outcomes. Future studies should investigate the impact of these additional sources of variability. Specifically, it is possible that an improved E-field isosurface threshold value could be identified that would better match the experimental data used in our analyses. However, neural polarization from extracellular stimulation is known to be related to the derivative of the E-field, and not the E-field itself [69,70]. This makes any E-field isosurface an inherently flawed metric for VTA calculation, and this strategy will always be compromised in its ability to provide an accurate and robust predictor function [71]. Therefore, the most practical future direction is not necessarily to equalize all parameters and methodological components across the different approaches. Each of these parameters has distinct contextual meaning and functionality depending on whether the method is VTA-based or DF-based. A suggested strategy for the future is to identify the optimal model parameters for each methodology, enabling each to achieve its best predictive performance.

### 4.3. Optimal DBS computational model may depend on specific application

Although we have demonstrated that using models with more realistic biophysical details in patient-specific space with pathway anatomical representation results in higher accuracy, this does not imply that other methodologies lack utility. The appropriate methodology depends on the specific application and the required level of accuracy. For example, the VTA-Structure method in patient-specific space showed reasonable performance (median r^2^ = 0.46) for HDP activation; however, for CSBT, its performance dropped significantly (median r^2^ = 0.31). Therefore, the selection of a methodology should consider, first, whether the focus is on therapeutic or side-effect pathways (or more generally near versus far pathways), and second, the degree of precision required for the specific clinical or research objective [72]. Furthermore, in some cases, patient-specific data are unavailable or unsuitable for group-level analyses [72], requiring the use of normative atlases to restore missing information. While studies attempting to correlate model-defined “sweet spots” with clinical outcomes have reported low correlation coefficients [12,15,57], they nonetheless achieved statistical significance. Additionally, sweet-spot targeting remains a common practice in clinical DBS, where structure -based methodologies have proven their utility [4,73]. Using VTA-based stimulation parameter selection has also been shown to diminish the cognitive and cognitive–motor declines associated with STN DBS [6]. However, it is noteworthy that such clinical improvement might also be mediated by correlating DBS lead locations rather than model-based activation estimates with best outcomes.

It is also noteworthy that when using the F1 score metric—focused on the presence of fiber activation rather than the degree of fiber activation —DF-Native-Pathway lost its superiority, with no significant difference between the methodologies (except when compared to VTA-Normative-Pathway, which performed worse than the others). This suggests that if the clinical goal is merely to predict the presence of CSBT activation side effects in a particular setting, most strategies can provide comparable predictive performance (assuming any internal capsule fiber activation results in a clinical side effect which is also unknown). However, if quantifying the degree of pathway activation is critical, DF-Native-Pathway remains the best option based on our findings.

## 5. Limitations

Our study has several limitations: 1) In implementing different models, we used two software packages and for consistency we used one structural and one pathway atlas. While these software programs capture the latest standards for implementing VTA and DF predictors, it is possible that our results are impacted by the specific tools we have chosen and, in the future, examining other alternatives could make the conclusions more generalizable. Additional sources of variability, including those related to electric field calculation and fiber diameters, were also not considered here. Specifically, in Lead-DBS, we used the SimBio/FieldTrip pipeline and standard GUI to input stimulation configurations, ensuring consistent VTA construction across all methodologies. Extending this work using other approaches to calculate and handle electric fields - such as OSS-DBS with non-binary or dynamic thresholds or pathway activation models (PAM), could represent a new methodology distinct from binary VTA. 2) Our statistical analysis was limited by relatively low number of patients which is a common limitation in intraoperative research. This may have affected results for CSBT pathway, where some activation measures were all zero either in-silico or in-vivo. 3) We focused on two specific pathways, while there are other clinically relevant pathways. A broader subthalamic pathway inclusion and activation measure could make the work more generalizable. 4) To keep DBS contact localization consistent across different models, we used localization tools in StimVision and then used the same coordinates for VTA modeling. While this localization matching helped compensate for discrepancies between the imaging pipelines used in DF- and VTA-based methods, it bypassed alternative localization and imaging strategies available in Lead-DBS, which could have influenced the results. Moreover, postoperative CT images were not available for most patients, which could have been used in Lead-DBS for automatic reconstruction of lead and contact localization. The dark artifact in postoperative T1-weighted MRI is generally more amorphous and disruptive to surrounding voxels than that in postoperative CT images. This may have affected our localization process. However, the use of postoperative T1 has been successful in our previous work [29], likely because it better conforms to the local anatomy around the lead compared to postoperative CT. 5) The DF and VTA methods of this study did not apply patient-specific anisotropy parameters into their activation estimation, but adding this feature could potentially make their predictions more accurate compared to ground truth. This has previously been done using custom modeling pipelines [50]. 6) It is worth noting that we used the default parameters in Lead-DBS for constructing VTA models including electric field threshold and white and gray matter conductivity, which may have influenced performance. While aligning parameters across the two modeling methods is challenging—given their different conceptual frameworks, future studies should identify optimal parameters for each methodology in relation to the specific application. 7) In this work, our validation of activation methods did not explicitly address the modeling of the electric field, because both VTA- and DF-based methods use the same electric field calculation, which was previously validated experimentally [74]. However, differences in FEM implementation could have impacted calculated field values.

## 6. Conclusion

While the DF-Native-Pathway model proved to be the most accurate method for quantitatively predicting experimental subcortical pathway activations, we believe that the choice of methodology should depend on the specific application and the required level of precision in clinical, surgical, or research settings. Our analysis showed that using normative brain space, instead of native (i.e., patient-specific) space, significantly diminished the accuracy of model predictions. Although the DF and VTA modeling methods exhibited comparable accuracy for the hyperdirect pathway, they diverged significantly in their predictions for the corticospinal tract, likely due to the VTA method’s limitations in estimating stimulation effects at greater distances from the electrode. These findings offer valuable guidance for developing more accurate models, facilitating reliable DBS outcome predictions, and advancing both clinical practice and scientific research.

## Supplementary Material

Supplementary

## Figures and Tables

**Table 1:** Patient demographics and experimental setup.

Patient code	Age/Sex	ECoG laterality at M1 (mm)	Number of monopolar DBS settings modeled	Number of bipolar DBS settings modeled	DBS lead	Lead type	DBS lead bottom contact MCP coordinates (mm)		
P01	62/M	34.0	14	22	MDT 3389	Standard	12.1	− 4.9	− 3.5
P02	53/M	40.9	14	28	MDT 3389	Standard	8.8	− 1.1	− 4.6
P03	57/M	24.3	14	25	MDT 3389	Standard	10.8	− 3.9	− 2.8
P04	44/M	18.9	33	9	ABT 6172	Steerable	9.8	− 4.9	− 5.9
P05	53/M	28.0	39	10	BSC 2202	Steerable	10.7	− 2.5	− 6.6
P06	60/M	26.5	34	6	ABT 6172	Steerable	−12.7	− 2.3	− 4.2
P07	64/M	35.0	0	18	MDT 3389	Standard	13.1	− 5.4	− 6.1
P08	65/M	44.0	14	20	MDT 3389	Standard	13.4	− 1.3	− 4.1
P09	60/M	41.5	0	18	MDT 3387	Standard	12.7	− 2.4	− 4.0
P10	69/M	27.5	9	18	BSC 2201	Standard	9.3	− 4.8	− 7.3
P11	67/M	22.6	0	15	MDT 3387	Standard	10.9	− 1.6	− 4.6

M1 = primary motor cortex; MCP = mid-commissural point; ABT = Abbott; BSC = Boston Scientific; MDT = Medtronic.

## Data Availability

The data that supports the findings of this study are available from the corresponding author, upon reasonable request.

## Appendix A. Supplementary data

Supplementary data to this article can be found online at https://doi.org/10.1016/j.brs.2025.10.022.
