# Supplementary material for: Evaluation of DBS computational modeling methodologies using in-vivo electrophysiology in Parkinson’s disease
Source: Brain Stimul. Author manuscript; Available in PMC 2025 Nov 24. (PMC12640560; doi:10.1016/j.brs.2025.10.022)
Supplement: Supplementary [file NIHMS2123602-supplement-Supplementary.docx]

# Supplementary Material

## Supplementary Methods

##### Neuroimaging and Lead Localization

Preoperative T1-weighted (T1w) MRI scans were obtained using 1.5T or 3T scanners, as detailed in ^1^. For DF-based models, the FMRIB Software Library (FSL) ^2^ was used for neuroimaging analysis. T1w images were corrected for RF/B1 inhomogeneities with FMRIB’s Automated Segmentation Tool (FAST) ^3^ and underwent skull stripping using FSL’s Brain Extraction Tool (BET) ^4^. Pre- and post-operative MRIs registration was performed using FSL’s FLIRT ^5,6^. In cases where FLIRT resulted in noticeable distortions in the ventricles or major anatomical structures—such as the cerebellum, corpus callosum, or large gray matter regions—we instead used FNIRT ^7^. Images were co-registered with a rigid-body affine transformation and Normalized Mutual Information as the cost function, unless registration was poor, in which case a twelve-parameter affine transformation was applied (for P01, P03, and P06).

For VTA-based models, the postoperative T1 were co-registered and then to match the reference preoperative T1 image resolution resliced and interpolated to the upsampled T1 using SPM 12 and Advanced Normalization Tools (ANTs) through built-in modules in Lead-DBS V3.1^8^. Co-registration results were then visually inspected using built-in tools. To compensate for deformation of the brain during surgery, we also applied the brain-shift correction method embedded in Lead-DBS ^8,9^.

The brain atlases used to estimate anatomical volumes or pathways within the imaging data were processed using a pipeline detailed in the Anatomical Representation section below. Postoperative T1-weighted images were used for lead localization. This choice was driven by the availability of postoperative MRI images across all patients (except P10, where postoperative CT was used instead).

For DF-based models, lead localization was performed using a custom MATLAB script with thresholding and orthogonal distance regression (ODR) ^1^. To make sure that DBS contact localization was consistent across different computational models, identified contact locations were marked (‘burned’) in the postoperative T1w images using 3D Slicer v5.2.1 and imported into Lead-DBS for VTA-based modeling. Using the manual lead localization tool in Lead-DBS, we then aligned the electrode contacts to the marked points marked in the postoperative T1w images. This approach ensured consistency in electrode positions between the DF-based and VTA-based modeling frameworks.

##### Customized algorithm to calculate VTA pathway intersection

We first converted the VTA NIfTI file format produced in Lead-DBS into a graphical object with surfaces and vertices using *isosurface* MATLAB function. Then for each point defining a fiber trajectory, we checked whether it resided inside the VTA object using *inpolyhedron* function. If at least one point was located inside the VTA, that fiber was considered to be activated.

##### Customized algorithm to calculate VTA volume overlap in native space

For the overlaps in the native space, we developed a custom MATLAB code where we calculated the percentages of the structure (IC or STN) vertices located inside VTA objects in response to each DBS setting (as detailed in the supplementary material). The rationale behind this approach was that since the voxel size was the same for the VTA and structures, the ratio of overlapped vertices should be similar to the ratio of overlapped volumes (voxels). To ensure that our new overlap percentage calculation method in native space was consistent with the method used in Lead-DBS (available only for normative models), we recalculated the overlap volume percentage in normative space using the custom code and correlated with Lead-DBS overlap values. Across 5 patients (P01-05), the average correlation coefficients between the two methods were 0.990 ±0.008 and 0.916 ±0.117 for STN and IC structures, respectively.

##### Directional vs non-directional setting comparison

In three patients with directional leads, for HDP, in Non-Directional settings, the R^2^ was 0.75 (IQR: 0.73-0.82) for DF compared to 0.59 (IQR: 0.57-0.61) for VTA. In directional settings, these values reduced to 0.51 (IQR: 0.47-0.56) for DF compared to 0.48 (IQR: 0.47-0.69) for VTA. For CSBT, in Non-Directional settings, the R^2^ median was 0.85 (IQR: 0.71-0.89) for DF compared to 0.41 (IQR: 0.23-0.42) for VTA. In directional settings, these values changed to 0.60 (IQR: 0.57-0.62) for DF compared to 0.43 (IQR: 0.41-0.47) for VTA. Accordingly, in DF method, in both pathways, the performance medians reduced when only directional settings were considered. In VTA method, while in HDP the median reduced for directional settings, it slightly improved in CSBT.

## Supplementary Tables and Figures

**Table S1-**Performance comparison of all the methodologies in predicting HDP activation

| HDP | Native space | | | Normative space | | |
| --- | --- | --- | --- | --- | --- | --- |
|  | DF -Pathway | VTA -Volume | VTA -Pathway | DF- -Pathway | VTA- -Volume | VTA-Pathway |
| P01 | 0.765 | 0.236 | 0.131 | 0.687 | 0.163 | 0.310 |
| P02 | 0.363 | 0.544 | 0.462 | 0.335 | 0.551 | 0.443 |
| P03 | 0.856 | 0.624 | 0.540 | 0.879 | 0.569 | 0.573 |
| P04 | 0.659 | 0.459 | 0.369 | 0.646 | 0.451 | 0.419 |
| P05 | 0.641 | 0.802 | 0.568 | 0.584 | 0.774 | 0.771 |
| P06 | 0.465 | 0.458 | 0.382 | 0.498 | 0.398 | 0.412 |
| P07 | 0.812 | 0.475 | 0.179 | 0.658 | 0.392 | 0.606 |
| P08 | 0.672 | 0.232 | 0.635 | 0.489 | 0.110 | 0.487 |
| P09 | 0.725 | 0.602 | 0.604 | 0.459 | 0.635 | 0.749 |
| P10 | 0.878 | 0.432 | 0.754 | 0.250 | 0.317 | 0.392 |
| P11 | 0.799 | 0.381 | 0.618 | 0.206 | 0.360 | 0.321 |
| Median | 0.725 | 0.459 | 0.540 | 0.498 | 0.398 | 0.443 |
| Q1 | 0.646 | 0.394 | 0.394 | 0.366 | 0.328 | 0.397 |
| Q3 | 0.809 | 0.588 | 0.588 | 0.655 | 0.565 | 0.598 |
| Mean | 0.694 | 0.477 | 0.476 | 0.517 | 0.429 | 0.499 |
| SD | 0.160 | 0.167 | 0.195 | 0.202 | 0.197 | 0.158 |

**Table S2-** Performance comparison of all the methodologies in predicting CSBT activation

| CSBT | Native space | | | Normative space | | |
| --- | --- | --- | --- | --- | --- | --- |
|  | DF -Pathway | VTA -Volume | VTA -Pathway | DF -Pathway | VTA- -Volume | VTA-Pathway |
| P01 | 0.540 | 0.305 | NaN | 0.269 | 0.000 | NaN |
| P02 | 0.406 | 0.093 | 0.000 | 0.398 | 0.039 | 0.001 |
| P03 | 0.586 | 0.384 | NaN | 0.419 | 0.229 | NaN |
| P04 | 0.687 | 0.374 | 0.000 | 0.523 | 0.346 | 0.086 |
| P05 | 0.589 | 0.319 | 0.000 | 0.488 | 0.248 | 0.123 |
| P06 | 0.720 | 0.531 | 0.212 | 0.720 | 0.478 | 0.092 |
| P07 | NaN | NaN | NaN | NaN | NaN | NaN |
| P08 | 0.822 | 0.002 | NaN | 0.367 | 0.000 | 0.002 |
| P09 | 0.036 | 0.030 | 0.000 | 0.065 | 0.042 | 0.041 |
| P10 | NaN | NaN | NaN | NaN | NaN | NaN |
| P11 | NaN | NaN | NaN | NaN | NaN | NaN |
| Median | 0.588 | 0.311 | 0.000 | 0.408 | 0.136 | 0.064 |
| Q1 | 0.473 | 0.0615 | 0.0615 | 0.318 | 0.0195 | 0.002 |
| Q3 | 0.7035 | 0.379 | 0.379 | 0.5055 | 0.297 | 0.092 |
| Mean | 0.548 | 0.255 | 0.042 | 0.406 | 0.173 | 0.058 |
| SD | 0.242 | 0.191 | 0.095 | 0.191 | 0.180 | 0.051 |


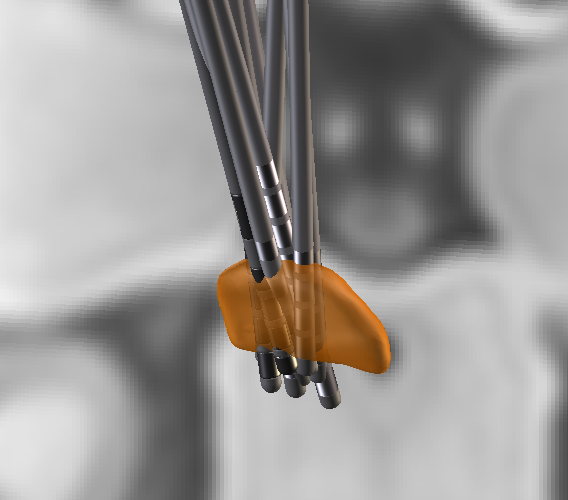


Figure S1- Visualizations of all leads implanted in the right hemisphere using Lead DBS v3.1. This includes all patients except P06 whose lead was implanted in the left hemisphere.


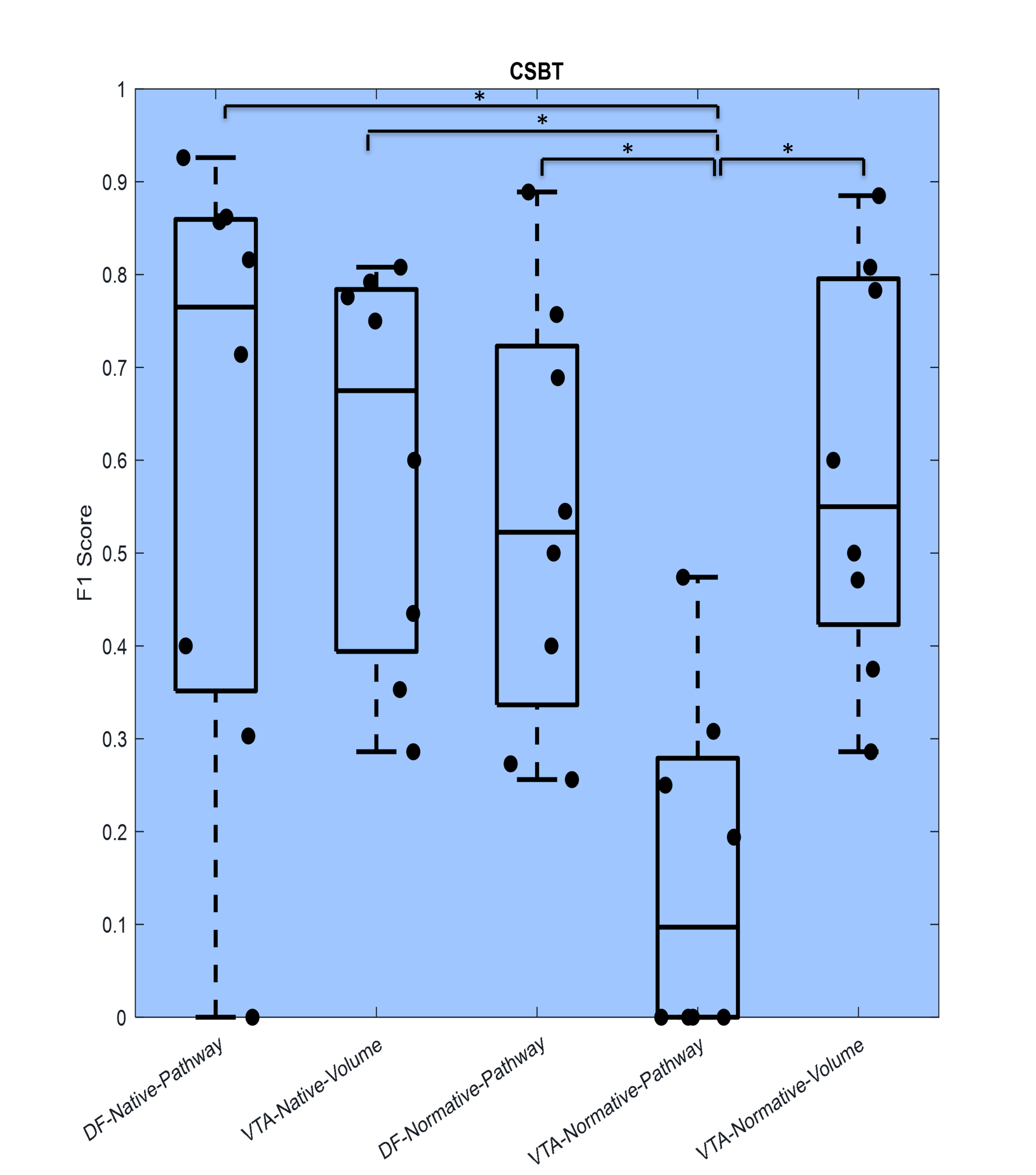


Figure S2- F1 Score comparison between the performances of all methodologies in CSBT. The pairs with significant differences are shown with bracket and asterisk (*).

Figure S3- Predictive performance comparison of two model types—DF-Native-Pathway and VTA-Normative-Structure—under directional and non-directional settings for three patients (P04, P05, P06) with directional leads. Pathways of interest: HDP (green, left) and CSBT (blue, right).

Figure S4- Comparison of VTA overlaps with STN and motor STN. Left: Correlation between the R² values of VTA–Normative–Structure using either STN or motor STN. Middle: Example of lead reconstruction for P03, where the motor STN and STN are shown in pink and orange, respectively, based on Distal atals. Right: Example of lead reconstruction for P03 shown with HDP M1 (green) division from Peterson atlas. (middle and right panels are illustrated in Lead DBS in MNI space).

## Supplementary references

1. Howell B, Isbaine F, Willie JT, et al. Image-based biophysical modeling predicts cortical potentials evoked with subthalamic deep brain stimulation. *Brain Stimul*. 2021;14(3):549-563. doi:10.1016/j.brs.2021.03.009

2. Smith SM, Jenkinson M, Woolrich MW, et al. Advances in functional and structural MR image analysis and implementation as FSL. *Neuroimage*. 2004;23 Suppl 1(SUPPL. 1). doi:10.1016/J.NEUROIMAGE.2004.07.051

3. Zhang Y, Brady M, Smith S. Segmentation of brain MR images through a hidden Markov random field model and the expectation-maximization algorithm. *IEEE Trans Med Imaging*. 2001;20(1):45-57. doi:10.1109/42.906424

4. Nakamura Y, Uematsu A, Okanoya K, Koike S. BET2 : MR-Based Estimation of Brain, Skull and Scalp Surfaces. *Eleventh Annual Meeting of the Organization for Human Brain Mapping, 2005*. 2005;17(10):167-. doi:10.1002/HBM.25843

5. Jenkinson M, Smith S. A global optimisation method for robust affine registration of brain images. *Med Image Anal*. 2001;5(2):143-156. doi:10.1016/S1361-8415(01)00036-6

6. Jenkinson M, Bannister P, Brady M, Smith S. Improved optimization for the robust and accurate linear registration and motion correction of brain images. *Neuroimage*. 2002;17(2):825-841. doi:10.1016/S1053-8119(02)91132-8

7. Andersson JLR, Jenkinson M, Smith S. Non-linear registration aka Spatial normalisation FMRIB Technial Report TR07JA2. Published online 2007.

8. Neudorfer C, Butenko K, Oxenford S, et al. Lead-DBS v3.0: Mapping deep brain stimulation effects to local anatomy and global networks. *Neuroimage*. 2023;268. doi:10.1016/j.neuroimage.2023.119862

9. Horn A, Li N, Dembek TA, et al. Lead-DBS v2: Towards a comprehensive pipeline for deep brain stimulation imaging. *Neuroimage*. 2019;184:293-316. doi:10.1016/J.NEUROIMAGE.2018.08.068
